# MCPH1, beyond its role deciding the brain size

**DOI:** 10.18632/aging.203658

**Published:** 2021-10-27

**Authors:** Martina Kristofova, Zhao-Qi Wang

**Affiliations:** 1 Leibniz Institute on Aging - Fritz Lipmann Institute (FLI), Jena 07745, Germany; 2 Faculty of Biological Sciences, Friedrich-Schiller University of Jena, Jena 07745, Germany

**Keywords:** MCPH1, BRCT domain, microcephaly, gonad development, tumorigenesis

The unusually large human brain with its greatly expanded cerebral cortex has attracted scientists from different disciplines. In order for this highly complex organ to develop, a delicate regulation of gene expression, migration and differentiation of neural stem cells is essential. In humans, billions of neurons are produced by mid-gestation in the process of neurogenesis. Firstly, the neural stem cell pool is expanded by what is called a “symmetric” mode of cell division. Later, neural stem cells undergo asymmetric division, which leads to the production of one neuroprogenitor and a committed neuronal precursor, or symmetric neurogenic division to produce two neurons. Among other molecular and cellular events, the precise control of cell cycle progression influences these two modes of divisions. Any perturbations of these mechanisms may lead to brain development disorders - a prominent example is human primary microcephaly (MCPH).

MCPH affects one in 30,000 to 250,000 people depending on a population, with a higher incidence in the populations where consanguineous marriages are common [1,2]. Generally, MCPH is characterized by a reduced brain size at birth with a mild degree of intellectual disabilities. Defects in other organs were only seldomly reported, if at all, suggesting that the brain is the main organ affected in MCPH patients. However, given that microcephaly in humans is a genetically heterogenous disorder with more than 25 genes involved, most of which play a role in general cellular processes, such as cell division apparatus [3,4], it is rather surprising that the mutations would manifest only in the brain.

**Figure 1:**
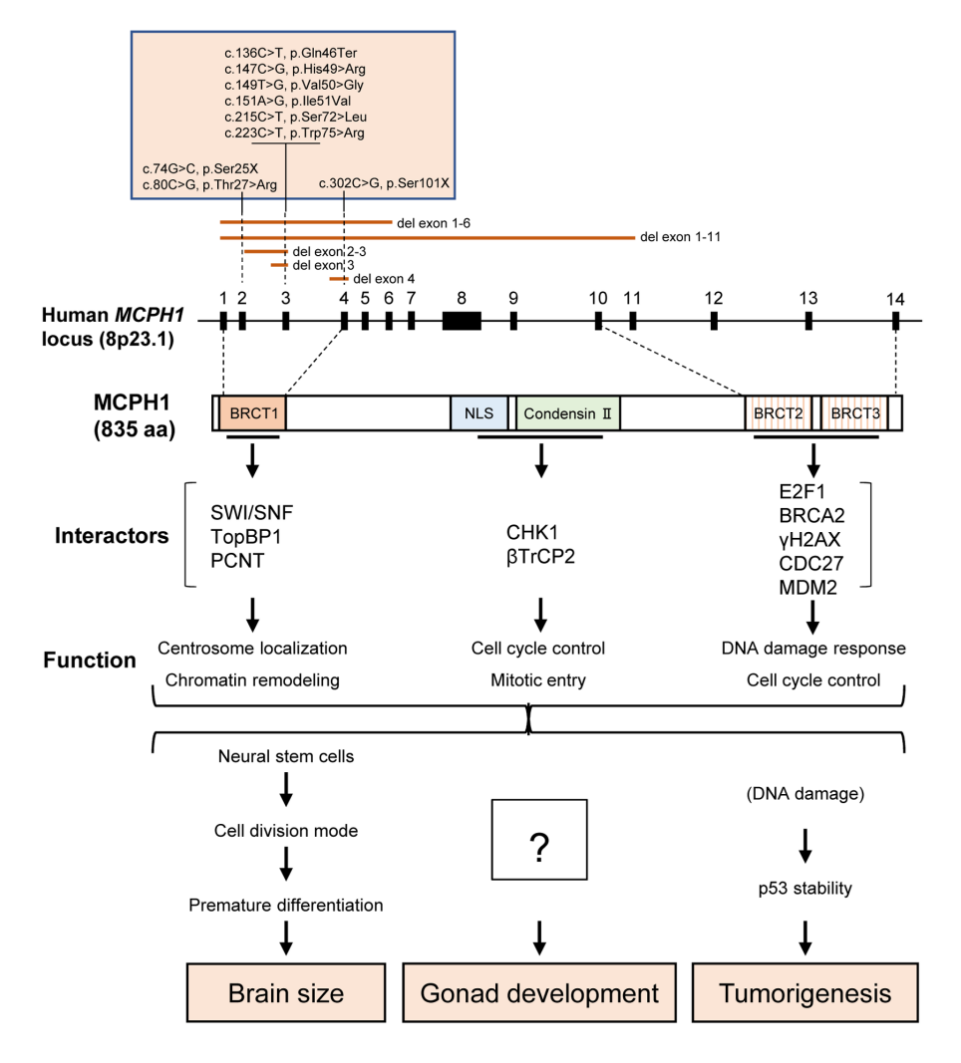
**Structure and function of MCPH1**. The majority of mutations are located in the N-terminus of MCPH1. Known interactors and their cellular functions are indicated. “c” stands for cDNA and “p” for protein sequence.

MCPH1 is the one of the most commonly mutated MCPH genes in primary microcephaly type 1 (MCPH1, OMIM251200) and is allelic to the premature chromosome condensation syndrome (PCC, OMIM 606858). This gene has been shown to be important in DNA damage response, cell cycle control and chromatin remodeling. Animal model studies have demonstrated the role of MCPH1 in brain development and PCC, as well as fertility [5]. It is well noted that most of the mutations are located in the N-terminus that comprises a BRCT domain, which typically interacts with phosphorylated proteins (Figure 1). This mutation spectrum strongly suggests an importance of the N-terminal domain in the manifestation of the disease. Our recent study [6] showed that a deletion of the N-terminal domain in a mouse model (MCPH1-ΔBR1), but retaining the rest of the MCPH1 protein, leads not only to microcephaly but also a gonad atrophy and infertility. Intriguingly, almost all female mutant mice develop ovarian tumors. Perhaps it is surprising to repetitively observe the infertility and gonad developmental defects [5–7] because these have not been reported in MCPH1 patients. The near 100% penetrance of ovarian tumors is striking, yet consistent with the notion that MCPH1 is down-regulated or mutated in human cancer patients [8], although MCPH1 patients have not been reported to be cancer prone. The MCPH1-ΔBR1 mouse model shows that the N-terminal BRCT domain is decisive for the physiological function of MCPH1 in brain size determination, and also in gonad development and tumor repression. Molecular and cellular studies have demonstrated that MCPH1 interacts with other partners via its mid and C-terminal domains for cell cycle control and tumorigenesis (Figure 1). Remarkably, the deletion of the N-terminal BRCT1 alone sufficiently recapitulates all pathologies reported for MCPH1 patients and various MCPH1 mouse models [6].

Many questions remain to be addressed. At a clinical genetic level, would human MCPH1 patients suffer fertility problems or be susceptible to cancer? At a molecular level, what makes the N-terminal BRCT1 domain of MCPH1 so important for particular organs (brain and gonads), but not others? It is plausible that the cell specificity is driven by cell type specific factors that interact with (the N-terminal) MCPH1 in a temporal-spatial manner. It is known that the N-terminal domain of MCPH1 is necessary for centrosome localization and for interaction with the chromatin remodeler SWI/SNF, which however could not necessarily explain the specific phenotypes of MCPH1 patients and mutant mouse models. Searching for novel partners that specifically interact with the N-terminal domain may uncover new molecular determinants in brain development and fertility, and perhaps also reveal new carcinogenesis pathways.
